# Ultrasonic-Assisted tumbling improves water retention and tenderness of wooden breast chicken meat

**DOI:** 10.1016/j.ultsonch.2025.107377

**Published:** 2025-05-22

**Authors:** Yanyan Lu, Zhenyang Wu, Tianjiao Bian, Xue Zhao

**Affiliations:** a State Key Laboratory of Meat Quality Control and Cultured Meat Development, Jiangsu Collaborative Innovation Center of Meat Production and Processing, Quality and Safety Control, Nanjing Agricultural University, Nanjing 210095, PR China; b School of Food and Biological Engineering, Hefei University of Technology, Hefei 230601, PR China

**Keywords:** Chicken, Wooden breast, Ultrasonic-assisted tumbling, Collagen, Quality improvement

## Abstract

Wooden breast myopathy results in reduced meat tenderness, poor water retention, and diminished processing value, underscoring the urgency to mitigate economic losses caused by the deteriorated quality of wooden chicken breast. This study evaluated the effects of ultrasound-assisted tumbling (400 W, 20 kHz, 80 min) on the quality characteristics of wooden breast meat with varying severity levels, involving normal (NB), moderate (MB), and severe (SB) breast. Results showed that ultrasound-assisted tumbling treatment significantly decreased (*P* < 0.05) shear force by 29.88 ± 0.23 %, 22.07 ± 0.28 %, and 19.41 ± 0.22 % in NB, MB, and SB samples, respectively, while reducing cooking losses by 22.98 ± 0.07 %, 13.81 ± 1.10 %, and 9.91 ± 0.27 %. Additionally, ultrasound-assisted tumbling significantly increased salt-soluble protein content (*P* < 0.05) through optimized protein-water interactions, thereby improving water-binding capacity. Meanwhile, the low-field NMR indicated that the immobilized-water proportion of MB increased (95.25 ± 0.45 %). While total collagen decreased from 2.92 mg/g to 2.79 mg/g and soluble collagen from 0.75 mg/g to 0.72 mg/g, no significant change was observed in collagen solubility (*P* > 0.05). This work firstly reports successful application of ultrasound-assisted tumbling to effectively improve water-holding capacity and tenderness of wooden breast meat, achieving treated MB comparable quality metrics to untreated NB.

## Graphical abstract

**Figure:**
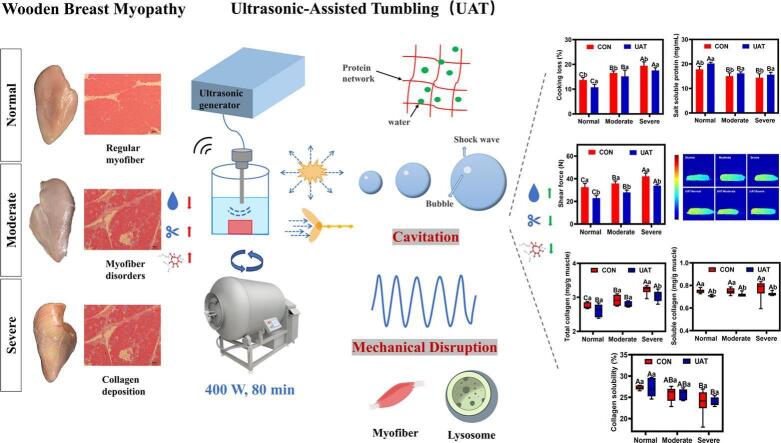

## 1 Introduction

Chicken is widely consumed globally owing to its nutritional advantages, including high protein content, low fat composition, and minimal cholesterol levels. To meet escalating market demands, the poultry industry has focused on enhancing broiler growth rates and muscle yields through selective breeding. However, this intensification has precipitated a marked increase in myopathic abnormalities, particularly wooden breast syndrome, in commercial broiler populations [1]. Wooden breast (WB), as a typical abnormal meat, has poor appearance, inferior eating quality, and low processing value characteristics, which brings huge economic losses to the poultry breeding and processing industries [2]. Clinical classification of WB severity employs standardized palpation protocols, typically categorizing lesions into three distinct grades based on tactile firmness and morphological alterations [3]. According to Xing et al. [4], the incidence of wooden myopathy in China was as high as 61.9 %, of which the incidence of moderate and severe wooden chicken breasts accounted for approximately 30.8 %. The marketability of poultry products depends fundamentally on quality parameters encompassing visual appearance, textural attributes, and flavor profiles [5]. WB meat exhibits pathognomonic white striations and mucoid exudates that significantly diminish consumer acceptance [6]. Histopathological analyses demonstrate distinct microstructural deviations between normal and wooden breast meat major muscles. While healthy myofibers maintain polygonal morphology with regular sarcomere alignment, WB meat displays disordered muscle architecture featuring mononuclear cell infiltration, degenerative myofibers, and ectopic lipid deposition [7].

Massive proliferation and excessive deposition of connective tissue are the most typical pathological features of wooden breast myopathy [6]. Excessive accumulation of the extracellular matrix and cross-linking of collagen fibers reduce the contractile elasticity and tenderness of meat, resulting in a harder texture [8]. Collagen is the most abundant protein in intramuscular connective tissue and plays a key role in determining meat texture. Elevated total collagen content coupled with reduced solubility indicates enhanced mature cross-link formation, particularly through lysyl oxidase-mediated intermolecular bonds [9]. These biochemical modifications increase collagen thermostability, thereby elevating shear force values and reducing meat tenderness [10]. Furthermore, myodegeneration and deposition of connective tissue and fat in wooden breast disrupt the water-holding capacity of meat proteins. Myofiber degeneration compromises the integrity of the fiber membrane, resulting in increased moisture loss and higher cooking losses [11]. Wooden breast myopathy alters the collagen profile in muscle by increasing total collagen content and reducing collagen solubility, significantly affecting the secondary structure content of proteins in connective tissue, extending the molecular structure of collagen, and causing protein disorder [12]. To improve the quality of wooden breast, various processing methods are being explored, among which tumbling marinating is a common approach in the meat industry.

Tumbling marinating is an essential step in the production process of western meat products, demonstrating superior efficacy over static marination through enhanced brine penetration, structural reorganization of myofibrillar networks, and accelerated ionic diffusion kinetics [13]. Studies have shown that tumbling marinating can improve marinade absorption, tenderness, and yield rate relative to conventional methods [14,15]. Nevertheless, process efficiency remains constrained by interdependent physical parameters, such as tumbling time, speed, temperature, and load capacity. Traditional single tumbling methods prove inadequate in satisfying consumer demands due to their low marinating efficiency and uneven improvement of meat quality. Consequently, vacuum-assisted tumbling methods are gradually dominating in meat processing, such as vacuum tumbling (VT) [16], pulsed vacuum tumbling (PVT) et al. [17]. However, there are non-negligible factors such as curing efficiency, temperature, oxidation, and vacuum efficiency, which lead to risks such as low efficiency, elevated meat temperatures, microbial growth, color deterioration, and excessive moisture loss [18]. Ultrasonic technology has been widely used in the meat processing industry for marinating, thawing, tenderizing, and sterilizing [19]. Ultrasound can increase membrane permeability and promote the penetration and diffusion of electrolyte ions, thus greatly improving the marinating efficiency [20]. Moreover, ultrasonic cavitation disrupts collagen fibrils and enhances solubilization, potentially counteracting the fibrotic characteristics of wooden breast.

Ultrasonic-assisted tumbling (UAT) is an emerging technology that synergizes ultrasonic cavitation (20–40 kHz) with mechanical tumbling to enhance marination efficiency. Unlike conventional methods, UAT leverages acoustic cavitation to disrupt collagen fibrils and increase membrane permeability, while tumbling mechanically loosens muscle structure [21]. This dual-action mechanism has been shown to improve the water retention of normal chicken breast, suggesting its potential to address the textural and moisture deficits characteristic of wooden breast. Owing to its strong penetrating power, ultrasound can form cavities in the medium. Upon the collapse of these cavities, high temperatures and pressures are released, which alter the muscle tissue structure and disrupt cell integrity [7]. Under the mechanical action of tumbling, collisions between raw meat and between raw meat and equipment loosen the muscle structure, which alters muscle tissue microstructure and enhances brine diffusion [22]. Studies demonstrate that ultrasonic-assisted tumbling reduces marination time from several hours to minutes compared with conventional tumbling, while achieving more uniform salt distribution [14,23]. Furthermore, the ultrasonic cavitation effect induces partial hydrolysis of collagen and myofibrillar proteins [24], thereby reducing shear force and enhancing meat tenderness significantly [25]. Critically, ultrasonic-assisted tumbling exhibits superior microbiological safety, as cavitation-induced localized thermal, pressures and oxidative effects, which disrupt bacterial cell membranes and inhibit enzymatic activity [26]. It has reported a reduction of 60 % of the natural microflora (*L. delbrueckii and L. monocytogenes*) compared to conventional tumbling [27], attributed to a synergistic mechanical-ultrasonic effect. Overall, the combined effects of ultrasonic cavitation and mechanical tumbling act synergistically on raw meat to improve overall meat quality [28]. However, due to its high equipment requirements and limited availability of suitable devices, the research on ultrasonic-assisted tumbling technology is still in the initial stage.

The integration of novel processing technologies with traditional tumbling marination is increasingly being used in the meat industry. However, related research in the field of wooden breast improvement technology is limited; the role and quality impact of ultrasonic-assisted tumbling in wooden breast meat remain unclear. In this study, wooden chicken breast is taken as the research object to explore the influence of ultrasonic-assisted tumbling time on wooden breast quality, aiming to improve the problem of low tenderness, poor water retention, and low nutritional value of wooden chicken breast, and reduce economic losses in the poultry breeding and processing industries.

## 2 Methods and material

### 2.1 Materials

WB meat was procured from the local poultry industry (Yike, Jiangsu, China). Referring to the wooden chicken breast grading criteria of Tijare et al. [3], the chicken breast samples were classified into three groups: normal, which comprises flexible throughout chicken breast; moderate, which comprises chicken breast hard throughout but flexible in the mid-to-caudal region; and severe, which comprises meat that is extremely hard and rigid throughout with bruising and discharge on the surface. All chemicals and reagents used were of at least analytical grade.

### 2.2 Ultrasonic combined tumbling marinade treatment

A total of 48 fillets were selected and divided equally into six groups, which were set up as three control (without UAT) and three experimental groups (with UAT). Chicken breasts was uniformly marinated in a 2.0 wt% sodium chloride solution (brine) containing 0.3 wt% composite sodium phosphate (pH 6.2–6.3) at 4 °C. The blending proportion of chicken breast and brine was set as 100/30 (w/v). All analyses (shear force, collagen content, TPA) were performed in triplicate for each sample (n = 6 × 8 × 3 replicates). Breast samples (CON, UAT per group: NB, MB, SB) were cut in a direction parallel to the muscle fibers, and trimmed into standardized (4 cm × 1 cm × 1 cm; 50 ± 2 g) rectangles to ensure uniform ultrasound exposure, with each treatment performed in triplicate. The ultrasonic equipment (THC-30BQG) provided by the Jining Tianhua Ultrasound Electronic Instruments Co., Ltd., (Ji Ning, China), which integrates dual ultrasonic and mechanical agitation systems. It includes vacuum pump unit (−0.08–0 MPa), an ultrasonic probe unit and an ultrasonic controller unit (adjustable power 0–1000 W). Ultrasound-assisted tumbling (UAT) was performed using a high-intensity 20 kHz probe system integrated into a custom stainless steel tumbler (volume: ≥ 30 L). The specific parameters were set as follows: ultrasonic frequency of 20 kHz, vacuum inside the drum of −0.08 MPa, drum angle of 55°, rotational speed of 8 rpm/min, 4 °C, tumbling mode of intermittent tumbling, ultrasonic-assisted tumbling for 10 min, and intermittent tumbling for 10 min. The effects of different ultrasonic power (0 W, 200 W, 400 W, and 600 W) and ultrasonic tumbling time (40 min, 80 min, and 120 min) on the meat quality of chicken breast were studied through pre-experimental, and optimal ultrasonic treatment conditions (400 W, 80 min) were established.

### 2.3 The marinating absorptivity

The marinating absorptivity (MA) was measured following the approach of Zhou et al. [29] with slight modifications. The formula for marinating absorptivity was as follows:

(1) $$\mathrm{MA}\left(\%\right)=\frac{{\text{M}}_{\text{2}}\text{-}{\text{M}}_{\text{1}}}{{\text{M}}_{\text{1}}}\times 100$$

The mass of chicken breast was recorded as M_1_ before ultrasonic-assisted tumbling. Then, the marinade on the surface of the chicken breast was wiped off using filter paper, and the mass of chicken breast was recorded as M_2_ after ultrasonic-assisted tumbling.

### 2.4 The salt-soluble protein content

The determination of salt-soluble protein content was conducted as described by Cofrades et al. [30] with slight modifications. First, 2.0 g of chicken breast meat was minced and mixed with 10 mL of pre-cooled PBS buffer (0.6 M KCl, 20 mM K_2_HPO_4_, 20 mM KH_2_PO_4_, pH 7.0), and homogenized at the speed of 12000 rpm for 1 min (20 s pause, 20 s on). Next, centrifugation was carried out (4 °C, 27,000 g for 30 min) to obtain a supernatant. Then, the protein concentration was determined according to the bicinchoninic urea method [31], using bovine serum protein (BSA) as the standard protein. The protein concentration was finally expressed as mg/mL.

### 2.5 Cooking loss

Cooking loss were measured according to Xing et al. [4] with slight modifications. Firstly, chicken breasts were cut into moderate shapes and weighed (weight was recorded as M_1_), then packed into cooking bags, and heated in a water bath at 75 °C until the center temperature reached 70 °C. After the cooking process, the samples were quickly cooled to room temperature with running water, wiped with filter paper to remove excessive water, and weighed again, weight was recorded as M_2_.

the cooking loss was calculated as follows:

(2) $$\mathrm{Cooking}\;\mathrm{loss}\left(\%\right)=\frac{{\text{M}}_{\text{1}}\text{-}{\text{M}}_{\text{2}}}{{\text{M}}_{\text{1}}}\times 100$$

M_1_ is the weight of chicken breasts before cooking, and M_2_ is the weight of chicken breasts after cooking.

### 2.6 Moisture distribution

Moisture distribution was measured according to Xing et al. [32]. First, the chicken breast was trimmed into a regular-shaped meat sample (1 cm × 1 cm × 3 cm), the moisture distribution was determined using the Carr-Purcell-Meiboom-Gill (CPMG) sequence (measurement temperature 32 °C, TW = 4000 ms, SW = 100 kHz, TE = 300 μs, NS = 32, and NECH = 3200). After collecting the data, the relaxation time T_2_ of the samples was analyzed using NMR analysis measurement software, and the inverse calculation was carried out using SIRT with an iteration number of 1,000,000 to obtain the transverse relaxation curves.

### 2.7 NMR transverse relaxation (T_2_) measurements

The low-field NMR spin–spin relaxation measurements were evaluated using a Niumag Pulsed NMR analyzer (PQ001; Niumag Corporation, Shanghai, China) following the method of Xing et al. [32]. A regular-shaped muscle sample (3 cm × 1 cm × 1 cm) was taken from each fillet for testing. The analyzer was maintained at a temperature of 32 °C under a resonant frequency of 22.4 MHz and a scanning frequency of 32 MHz. The transverse relaxation time (T_2_) was determined by utilizing the Carr-Purcell-Meiboom-Gill (CPMG) sequence with a τ-value of 300 µs. Data from 3200 echoes were collected through 32 scan repetitions and fitted using the program Multi Exp Inv Analysis.

### 2.8 Shear force

A total of 48 fillets were selected and divided equally into six groups, which were set up as three control (without UAT) and three experimental (with UAT). Shear force were performed in three replications for each sample (n = 6 × 8 × 3 replicates). Firstly, packed the sample into cooking bags, and heated in a water bath at 75 °C until the center temperature reached 70 °C. After the cooking process, the samples were quickly cooled to room temperature with running water, wiped with filter paper to remove excessive water. Then cut in a direction parallel to the muscle fibers trimmed into rectangles (4 cm × 1 cm × 1 cm, 50 ± 2 g), with each sample was cut into triplicates, and placed on the texture analyzer stage for triplicate measurement. After the lowest and highest peak force value were excluded, the average was recorded as the cutting force value for each sample. The cutting force was expressed as Newtons (N).

### 2.9 Texture profile analyses (TPA)

Meat samples of two cylinders (diameter: 20 mm, height: 20 mm), perpendicular to the direction of muscle fiber, were cut for texture profile measurement with a texture analyzer (XT Plus, Stable Micro Systems Ltd., Godalming, UK) according to Dondero et al. [33]. The measurement parameters were set as follows: two compression cycles with 50 % compression, trigger force of 5 g; and pre-test, test, and post-test speeds of 5, 5, and 10 mm/s, respectively. Each sample was measured three times.

### 2.10 Collagen profiles measurements

The Breast samples (n = 8, per group: NB, MB, SB) were set up as three control (without UAT) and three experimental groups (with UAT). The collagen content were performed in three repetitions for each sample (n = 6 × 8 × 3 replicates). The total collagen content analysis was measured according to Latorre et al. [10]. 4.0 g of the sample was hydrolyzed in 30 mL of sulfuric acid (3 M) for 16 h at 105 °C. The hydroxyproline content was calculated using a conversion ratio of 7.25 and subsequently reported as a percentage. The content of total collagen and soluble collagen content was determined by the method of Nishimura et al. [34]. Specifically, the 4.0 g of samples were homogenized in 8 mL of 1/4 Ringer’s solution at 12000 rpm for 40 s by a homogenizer (Ultra Turrax T-25 Basic, IKA, Staufen Germany). The homogenates underwent heating for 1 h at 77 °C, followed by centrifugation for 20 min at 4000 g. The amount of soluble collagen in the combined supernatant was detected utilizing the aforementioned procedures. Collagen solubility is defined as the ratio of heat-soluble collagen to the total amount of collagen.

### 2.11 Statistical analysis

All experiments were performed in triplicate at least for each, and all the measured data were analyzed by SPSS 26.0 statistical software. The effects of ultrasound power, processing time, and the interaction between the two on each index were analyzed by mixed model according to Duncan’s multiple range test. Significance was determined at *P* < 0.05, and the results are reported as mean ± standard deviation of the mean. The NMR images were obtained by using the imaging software that came with the system, and the pseudo-color images were obtained by using the Newmark MRI image processing software.

## 3 Results and discussion

### 3.1 Marinating absorptivity

**Fig. 1:**
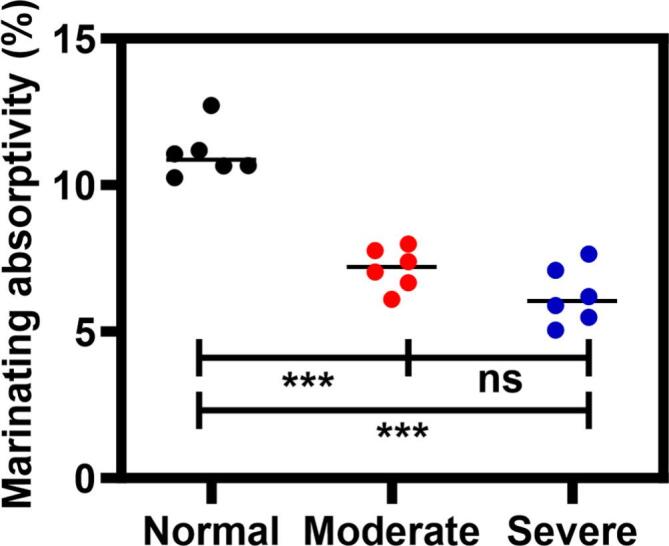
Effect of wooden breast myopathy on marinating absorptivity. *denote ultrasonic treatment effects: *P < 0.05, **P < 0.01, ***P < 0.001. Data are expressed as mean ± SD (n = 6), error bars indicate the standard deviations. Experimental groups: Normal: Normal breast; Moderate: Moderate wooden breast; Severe: Severe wooden breast.

Marination absorption capacity serves as a critical quality indicator for cured meat products, reflecting both preservation efficacy and textural enhancement potential [35]. Fig. 1 shows that wooden breast myopathy significantly influenced marinade absorption (*P* < 0.05). Compared to normal breast, the decrease (from 11.27 % to 6.23 %) in marinating absorptivity of severe wooden breast may be attributed to changes in its composition, primarily the increase in fat content and the accumulation of connective tissue. These structural modifications promote macrophage infiltration and disrupt adipocyte distribution patterns, culminating in elevated shear modulus that impedes brine penetration through reduced interstitial porosity, which is not conducive to capturing more brine during the curing process [8]. The marination absorption rate decreased with increasing severity of wooden breast. However, the marination absorptivity of chicken breasts with severe wooden breast was lower than that of moderate wooden breast (6.23 % vs 7.16 %), although no significant difference was observed between the two groups (*P* > 0.05). Maxwell et al. [36] also found that marinade absorptivity in tumbling-treated wooden chicken breasts was significantly lower than in normal meat. Ultrasound intervention (20–40 kHz) disrupted collagen stability of wooden breasts through fibril disorganization, loosening of fiber alignment, and triggering degeneration and granulation of collagen fiber in the extracellular space [37]. These changes can affect both the cavitation effect and the mechanical action of tumbling, ultimately resulting in reduced marinating absorptivity of chicken breast [11]. Notably, ultrasonic-assisted tumbling treatment has the potential to shorten marinating time from 8-10 h to 45–60 min through cavitation-induced microjet formation, which attacks myofibrils and generates microchannels in the meat, facilitating the penetration of NaCl into the tissue [38].

### 3.2 Salt-soluble protein content

**Fig. 2:**
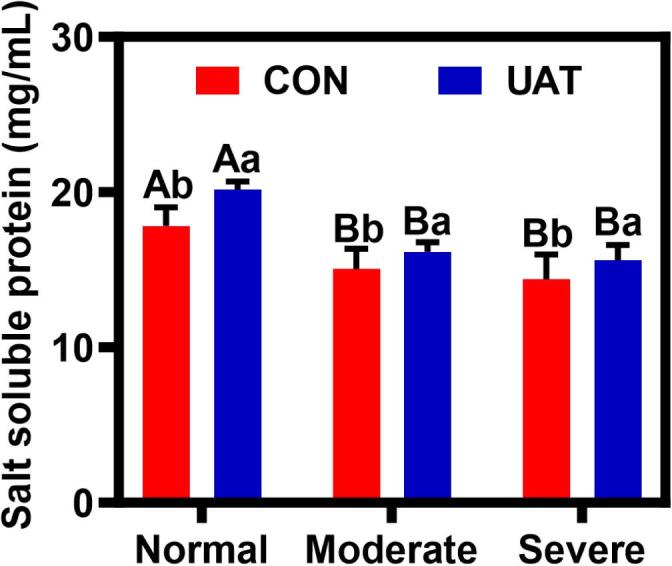
Effect of ultrasonic-assisted tumbling on salt soluble protein of wooden breast. A-B different letters indicate statistically significant difference between different wooden breast (*P* < 0.05); a-b different letters indicate statistically significant difference between different treatment (*P* < 0.05); CON: control; UAT: ultrasonic-assisted tumbling (400 W, 20 kHz, 80 min); Normal: Normal breast; Moderate: Moderate wooden breast; Severe: Severe wooden breast.

The salt-soluble protein content is a vital index for assessing the functional properties of proteins in muscle [39]. Myofibrillar proteins, which are solubilized at higher salt concentrations (> 0.3 mol/L), are classified as salt-soluble proteins. Previous studies have demonstrated that myofibrillar proteins bind Cl^–^ ions, thereby increasing electrostatic repulsion between protein molecules and promoting solubilization. Consequently, NaCl exhibits a dissolving effect [40]. As illustrated in Fig. 2, wooden breast myopathy significantly affected the amounts of salt-soluble proteins (*P* < 0.05), and the quantity of salt-soluble proteins decreased with increasing severity of the myopathy. However, no significant difference was observed between moderate and severe wooden breasts (*P* > 0.05). Normal chicken breasts exhibited a salt-soluble protein content of up to 18 mg/ml, whereas wooden chicken breasts contained only 14 mg/ml. This difference is due to myofiber degeneration compromising sarcolemmal integrity in WB tissue, facilitating endogenous protease release from the muscle fibers, resulting in the disorganization of structural proteins and protein hydrolysis reaction [41]. Additionally, protein denaturation due to inflammatory response and oxidative stress triggered by wooden breasts may be another cause [12]. After ultrasonic and tumbling, a significant increase in salt-soluble protein content was noted, 10.20 ± 0.65 %, 7.97 ± 0.48 %, and 8.47 ± 0.38 % (*P* < 0.05) in NB, MB, and SB samples, respectively (Fig. 2). Nevertheless, differences in the rate of increase between wooden and normal breasts remained evident. Unlike normal breast tissue, the tissue of wooden breast may require higher ultrasonic energy to achieve similar improvements [42]. Similar results have been reported by McDonnell et al. [43], who concluded that increased ultrasonic power and tumbling time enhance the extraction of salt-soluble proteins, an effect attributed to the mechanical action of tumbling. It is possible that ultrasound-assisted tumbling disrupts the muscle tissue structure and releases more salt-soluble proteins, which to some extent can promote the enrichment of salt-soluble proteins towards the meat surface, thus preventing water loss [44]. Notably, UAT treatment protein surfaces exhibited higher hydrophilic group exposure, enhancing water-binding capacity and reducing cooking losses. This interfacial modification mechanism explains the inverse correlation between salt-soluble protein content and moisture retention.

### 3.3 Cooking loss

**Fig. 3:**
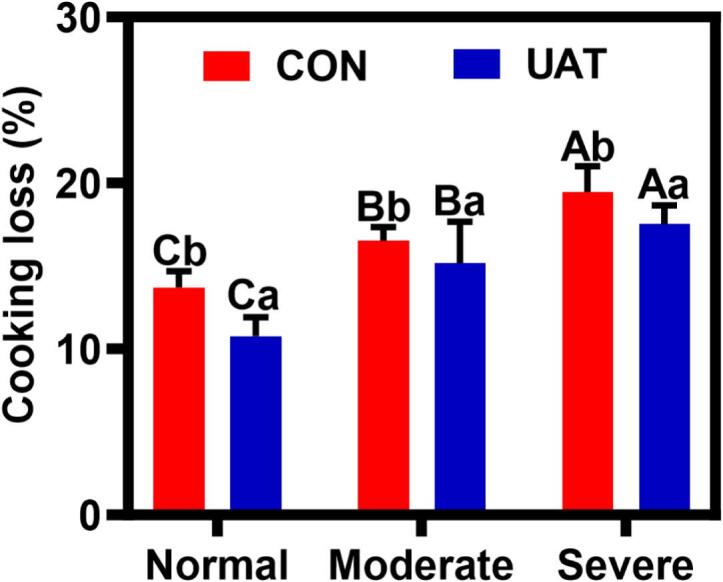
Effect of ultrasonic-assisted tumbling on cooking loss of wooden breast**.** A-C different letters indicate statistically significant difference between different degree of wooden breast condition (*P* < 0.05); a-b different letters indicate statistically significant difference between different treatment (*P* < 0.05); CON: control; UAT: ultrasonic-assisted tumbling (400 W, 20 kHz, 80 min) Normal: Normal breast; Moderate: Moderate wooden breast; Severe: Severe wooden breast.

Cooking loss is one of the most important indicators of water-holding capacity (WHC) in meat quality [45]. As Fig. 3 shows, wooden myopathy significantly increases cooking loss (*P* < 0.05), and the cooking loss was observed to increase with the increasing severity of wooden myopathy. This finding is consistent with previous studies showing that the wooden breast myopathy increases the cooking loss and reduces water retention [46]. This phenomenon may be attributed to myofibril degeneration along with the enhanced deposition of intramuscular connective tissue (IMCT) and adipose tissue, which disrupts the water-holding capacity of muscle proteins [11]. Additionally, the degradation of muscle fibers impairs the integrity of the fiber membrane, resulting in water loss during cooking [9]. Compared with untreated chicken breast, the cooking loss significantly decreased by 22.98 ± 0.07 %, 13.81 ± 1.10 %, and 9.91 ± 0.27 % (*P* < 0.05) in NB, MB, and SB samples, respectively (Fig. 3). Ultrasound treatment can enhance water retention by reducing protein particle size and exposing active regions, thereby increasing protein-water interactions and improving the water holding capacity [47]. Moreover, the cooking loss remained highest in severe breast and decreased in the order of moderate and normal chicken breast with a significant difference (17.53 % vs 15.16 % vs 11.06 %, *P* < 0.05). Previous studies have revealed that the 20 kHz ultrasonic frequency promoted cavitation-induced microchannel formation in wooden breast tissue [38], facilitating water migration, and the collapse of ultrasonic cavitation bubbles generates localized pressures exceeding 100 MPa, disrupting myofibrillar protein networks and enhancing water mobility in wooden breast [48].

### 3.4 Water mobility and distribution

The T_2_ relaxation time is a critical index for evaluating changes in moisture distribution [49]. As shown in Table 1, the T_2_ relaxation times (T_21_, T_22_, and T_23_) of the wooden breast exhibited distinct changes after ultrasonic-assisted tumbling treatment compared to the normal breast. Specifically, Severe WB muscles had longer relaxation times of T_21_ (2.37 ± 0.52 ms vs 1.65 ± 0.21 ms) and T_22_ (49.56 ± 2.89 ms vs 46.79 ± 1.47 ms), compared with the normal muscle (*P* < 0.05). Compared to the control group, T_21_ was significantly left-shifted in wooden breast, indicating a notable reduction in bound water, but no significant changes were observed in T_22_ and T_23_ (*P* > 0.05). Meanwhile, post-cooking analysis (Table 2) revealed a significant leftward shift in T_22_, substantially affecting the moisture distribution in WB samples (*P* < 0.05).

**Table 1:** Effect of ultrasonic-assisted tumbling treatment on water mobility and distribution of wooden breast.

Items	Group	Category		
		Normal	Moderate	Severe
T_21_(ms)	CON	1.65 ± 0.21^Ca^	2.03 ± 0.49^Ba^	2.37 ± 0.52^Aa^
	UAT	1.72 ± 0.16^Ba^	1.83 ± 0.20^Ba^	1.99 ± 0.21^Ab^
T_22_(ms)	CON	46.79 ± 1.47^Ba^	50.10 ± 4.09^Aa^	49.56 ± 2.89^Aa^
	UAT	47.57 ± 2.33^Ba^	49.40 ± 1.79^Aa^	49.74 ± 1.77^Aa^
T_23_(ms)	CON	229.51 ± 15.85^Aa^	227.85 ± 20.13^Aa^	236.05 ± 17.97^Aa^
	UAT	231.63 ± 15.06^Aa^	237.03 ± 10.98^Aa^	232.38 ± 10.23^Aa^
P_21_(%)	CON	1.90 ± 0.23^Aa^	1.72 ± 0.30^Bb^	1.60 ± 0.19^Bb^
	UAT	2.03 ± 0.25^Aa^	2.03 ± 0.24^Aa^	2.09 ± 0.29^Aa^
P_22_(%)	CON	96.08 ± 0.57^Aa^	94.47 ± 0.81^Ba^	94.07 ± 0.86^Ba^
	UAT	96.10 ± 0.66^Aa^	95.25 ± 0.45^Ba^	94.55 ± 0.68^Ca^
P_23_(%)	CON	2.02 ± 0.54^Ba^	3.81 ± 0.79^Aa^	4.33 ± 0.95^Ab^
	UAT	1.79 ± 0.58^Ca^	2.72 ± 0.49^Bb^	3.36 ± 0.76^Aa^

A-C different letters in the same row indicate statistically significant difference (*P* < 0.05); a-b different letters in the same column indicate statistically significant difference (*P* < 0.05); CON: control；UAT: ultrasonic-assisted tumbling (400 W, 20 kHz, 80 min). Normal: Normal breast; Moderate: Moderate wooden breast; Severe: Severe wooden breast.

**Table 2:** Effect of ultrasonic-assisted tumbling treatment on water mobility and distribution of cooked wooden breast.

Items	Group	Category		
		Normal	Moderate	Severe
T_21_(ms)	CON	1.73 ± 0.51^Aa^	2.07 ± 0.35^Aa^	1.93 ± 0.45^Aa^
	UAT	1.57 ± 0.26^Ba^	1.65 ± 0.29^Bb^	2.04 ± 0.52^Aa^
T_22_(ms)	CON	38.03 ± 1.72^Ba^	41.56 ± 2.36^Aa^	41.68 ± 3.99^Aa^
	UAT	37.96 ± 1.79^Aa^	36.63 ± 2.68^Ab^	38.52 ± 2.35^Ab^
T_23_(ms)	CON	472.77 ± 39.21^Ba^	592.76 ± 53.51^Aa^	568.29 ± 61.78^Aa^
	UAT	493.59 ± 48.57^Ba^	516.00 ± 46.24^Aa^	470.05 ± 45.61^Bb^
P_21_(%)	CON	0.99 ± 0.15^Aa^	0.90 ± 0.18^Bb^	0.91 ± 0.17^Aa^
	UAT	0.85 ± 0.12^Aa^	0.71 ± 0.12^Aa^	0.77 ± 0.14^Ab^
P_22_(%)	CON	95.88 ± 0.77^Aa^	95.98 ± 0.41^Ba^	95.73 ± 0.61^Ab^
	UAT	96.42 ± 0.47^Aa^	96.48 ± 0.44^Ba^	96.43 ± 0.33^Aa^
P_23_(%)	CON	3.09 ± 0.46^Ba^	3.15 ± 0.34^Aa^	3.62 ± 0.45^Aa^
	UAT	2.76 ± 0.51^Aa^	2.81 ± 0.37^Bb^	2.83 ± 0.21^Ab^

A-C different letters in the same row indicate statistically significant difference (*P* < 0.05); a-b different letters in the same column indicate statistically significant difference (*P* < 0.05); CON: control；UAT: ultrasonic-assisted tumbling (400 W, 20 kHz, 80 min). Normal: Normal breast; Moderate: Moderate wooden breast; Severe: Severe wooden breast.

As shown in Table 1, the proportion of bound water (P_21_) in wooden breast meat was significantly lower (*P* < 0.05), while free water (P_22_) was increased significantly (*P* < 0.05). However, no significant change was seen in the immobile water (*P* > 0.05). These findings corroborate previous research documenting analogous moisture distribution patterns [32]. Notably, ultrasonic intervention substantially reduced free water content by 12 % relative to untreated controls (*P* < 0.05), though values remained elevated compared to normal breast samples. These changes likely result from ultrasound-induced disruption of fibrotic collagen structures [50], facilitating the migration of intra-myofibrillar water to interstitial spaces. However, persistent free water excess suggests incomplete structural remediation, potentially attributable to sustained collagen cross-linking [12]. The increased cooking loss of wooden breast due to the changes in water mobility and distribution could potentially lead to economic losses in the meat industry.

Post-cooking analysis (Table 2) revealed reduced proportions of bound and immobile water in WB samples. However, ultrasonic-assisted tumbling treatment significantly enhanced immobile water content of MB (95.25 ± 0.45 %) (*P* < 0.05), indicating improved water retention capacity and reduced marination losses. Previous investigations have demonstrated that the percentage of free water in wooden chicken breast meat was significantly higher [51], which is in line with our observations. It is possible that wooden breast myopathy converted structural degradation of myofibrillar proteins, leading to water phase redistribution [52]. Overall, these observations suggest that compromised water-holding capacity appears intrinsically associated with myofibrillar protein reduction and altered moisture mobility patterns in wooden breast.

### 3.5 NMR

**Fig. 4:**
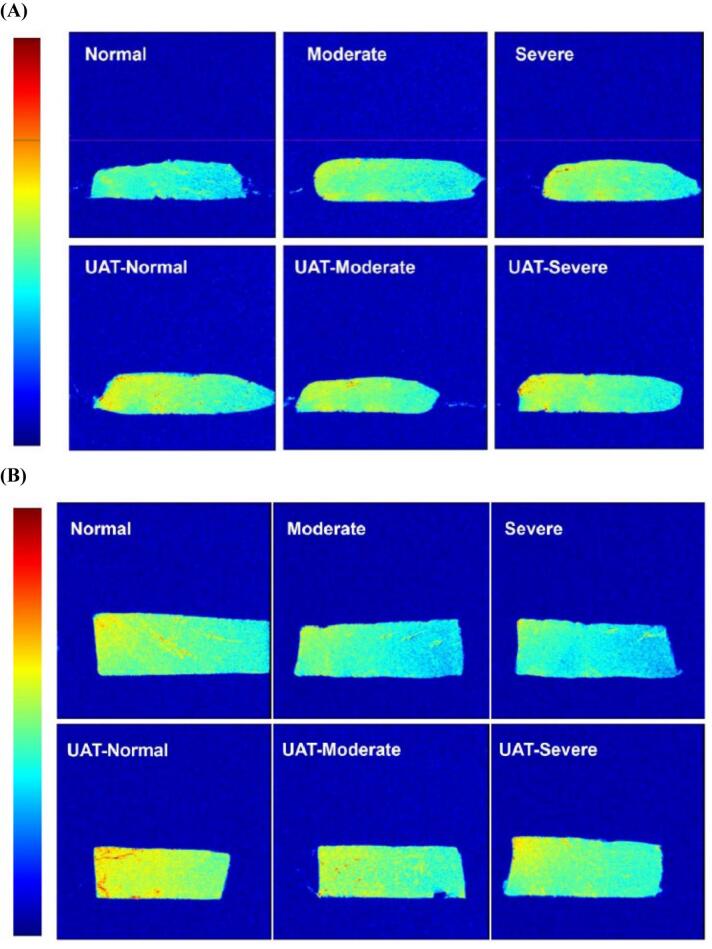
Nuclear magnetic resonance imaging of (A) wooden breast and (B) cooked, wooden breast after ultrasonic-assisted tumbling.

To further elucidate moisture dynamics, nuclear magnetic resonance (NMR) imaging analysis was employed for detailed characterization of wooden breast samples, as illustrated in Fig. 4A. This advanced analytical technique serves as a powerful tool for non-destructive assessment of moisture distribution in biological tissues, based on hydrogen ion signal detection that directly correlates with water content. Imaging analysis revealed a distinct pattern where the degree of wooden breast lignification negatively correlated with signal intensity, more severe lignification manifested as lighter image coloration, indicating reduced hydrogen ion signals, decreased water content, and consequently higher cooking losses. While untreated controls demonstrated minimal signal intensity, corresponding to compromised water retention, progressive image darkening was observed with increasing severity of wooden breast myopathy, suggesting a positive relationship between pathological lignification intensity and tissue moisture content. Notably, ultrasound-assisted tumbling treatment yielded significantly darker and more uniform image patterns compared to untreated samples (*P* < 0.05). This phenomenon may be attributed to enhanced marinade infusion into myofibrillar networks through ultrasonic cavitation effects, which facilitates both water retention improvement and spatial homogenization of moisture distribution within the meat matrix [42]. As demonstrated in Fig. 4B, cooked wooden chicken breasts exhibited higher moisture content than the control group, despite maintaining lighter coloration compared to normal breast samples. These observations suggest that ultrasonic-assisted tumbling treatment effectively enhances water retention capacity in meat products. The combined mechanical action of tumbling, which loosens muscle structure, and the synergistic collagen degradation effect of ultrasound treatment [53], address both textural and moisture-related defects characteristic of wooden breast myopathy. This dual mechanism of action presents a promising approach for mitigating quality deterioration in affected poultry products.

### 3.6 Shear force

**Fig. 5:**
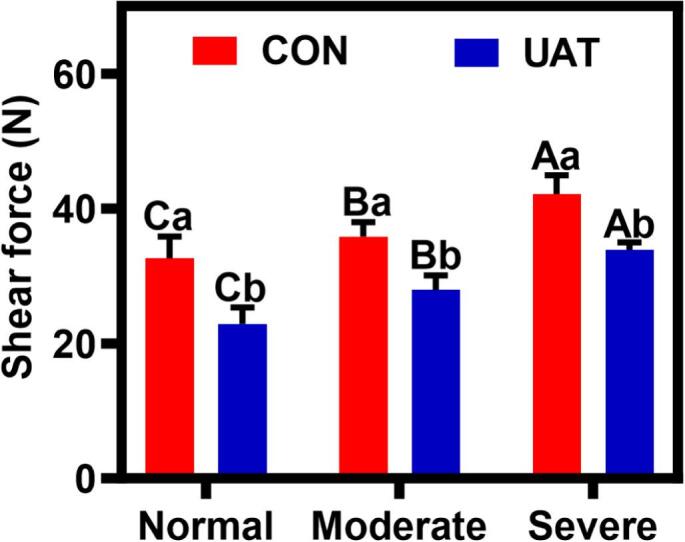
Effect of ultrasonic-assisted tumbling on shear force of wooden breast. A-C different letters indicate statistically significant difference between different degree of wooden breast condition (*P* < 0.05); a-b different letters indicate statistically significant difference between different treatment (*P* < 0.05); CON: control; UAT: ultrasonic-assisted tumbling (400 W, 20 kHz, 80 min); Normal: Normal breast; Moderate: Moderate wooden breast; Severe: Severe wooden breast.

Tenderness, a critical quality determinant influencing consumer acceptance of meat products, was quantitatively assessed through Warner-Bratzler shear force measurements. A higher shear force value indicates poorer tenderness, while a lower value indicates better tenderness [54]. As shown in Fig. 5, wooden breast exhibited significantly higher shear force values compared to normal breast (*P* < 0.05), and increased with the severity of wooden myopathy, which indicated that wooden myopathy would reduce the tenderness of meat. This inverse correlation between shear force and tenderness aligns with findings by Wang et al. [55], who identified lignification severity as a key modulator of tissue stiffness and shear resistance. Bian et al. [12] observed that wooden breast myopathy resulted in a deterioration of tenderness, with a higher shear force value of severe breast as compared with the normal meat (45.10 vs. 32.78; *P* < 0.05). A 37.6 % increase was attributed histologically to expanded interstitial connective tissue deposition [56]. Meanwhile, ultrasonic treatment significantly decreased shear force by 29.88 ± 0.23 %, 22.07 ± 0.28 %, and 19.41 ± 0.22 % in NB, MB, and SB samples, respectively (Fig. 5). Similarly, Chatterjee et al. [9] concluded that the shear force was higher in wooden breast meat compared with normal breast meat, attributing it to the accumulation of interstitial connective tissue. We hypothesize this tenderness decrease arises from elevated collagen content and altered molecular cross-linking patterns within the extracellular matrix. Severe wooden breast meat exhibits elevated collagen cross-linking [11], which reduces ultrasonic cavitation efficiency by increasing tissue rigidity. Moreover, the myodegeneration and the infiltration of adipose tissues in severe WB meat impairs acoustic impedance matching [12].

### 3.7 Textural properties

Texture is a critical parameter in meat quality assessment, with hardness representing the force required to achieve specific deformation or penetration of a food matrix. Gumminess is a secondary textural property that describes the energy required to disintegrate a semi-solid food product until it is ready for swallowing, while chewiness measures the mastication energy required for solid foods [54]. Our findings demonstrate that wooden breast myopathy significantly impacts textural properties, particularly hardness, adhesiveness, and chewiness, regardless of whether the meat was treated with ultrasound-assisted tumbling. From Table 3, distinct variations in hardness, gumminess, and chewiness were observed between normal and wooden breast samples (*P* < 0.05). However, no significant differences were observed regarding springiness, cohesiveness, and resilience (*P* > 0.05). Similar findings were reported by Zhang et al. [57], who indicated that elevated values for these parameters in wooden breast meat, with more pronounced differences in severe compared to moderate breast. The textural alterations in wooden breast myopathy are mechanistically linked to changes in extracellular matrix composition. The condition affects glycosaminoglycan levels, crucial for extracellular matrix formation, and core proteoglycans, which modulate collagen cross-linking [58]. Increased core proteoglycan concentrations promote tighter collagen fiber alignment, ultimately enhancing meat hardness.

**Table 3:** Effect of ultrasonic-assisted tumbling on TPA of wooden breast.

Items	Group	Category		
		Normal	Moderate	Severe
Hardness(g)	CON	2553.51 ± 224.06^Ba^	2799.64 ± 287.62^ABa^	3038.03 ± 464.70^Aa^
	UAT	1975.58 ± 133.69^Bb^	2119.54 ± 198.30^Bb^	2354.06 ± 278.01^Ab^
Springiness(mm)	CON	0.54 ± 0.06^Aa^	0.55 ± 0.06^Aa^	0.55 ± 0.06^Aa^
	UAT	0.56 ± 0.02^Aa^	0.57 ± 0.06^Aa^	0.56 ± 0.03^Aa^
Cohesiveness	CON	0.40 ± 0.05^Aa^	0.41 ± 0.04^Aa^	0.41 ± 0.05^Aa^
	UAT	0.39 ± 0.05^Aa^	0.4 ± 0.04^Aa^	0.41 ± 0.03^Aa^
Gumminess(g)	CON	1072.19 ± 196.41^Ba^	1182.27 ± 154.16^ABa^	1241.34 ± 123.21^Aa^
	UAT	726.13 ± 127.33^Bb^	850.78 ± 115.57^Ab^	966.29 ± 95.55^Ab^
Chewiness(g.mm)	CON	590.62 ± 67.33^Ba^	606.46 ± 55.37^Ba^	679.49 ± 51.81^Aa^
	UAT	458.71 ± 62.84^Bb^	549.65 ± 40.31^ABa^	535.36 ± 60.05^Ab^
Resilience	CON	0.20 ± 0.03^Aa^	0.20 ± 0.03^Aa^	0.20 ± 0.04^Aa^
	UAT	0.18 ± 0.02^Aa^	0.19 ± 0.02^Aa^	0.19 ± 0.02^Aa^

A-C different letters in the same row indicate statistically significant difference (*P* < 0.05); a-b different letters in the same column indicate statistically significant difference (*P* < 0.05); CON: control; UAT: ultrasonic-assisted tumbling (400 W, 20 kHz, 80 min). Normal: Normal breast; Moderate: Moderate wooden breast; Severe: Severe wooden breast.

Ultrasound-assisted tumbling treatment significantly improved the hardness, adhesiveness, and chewiness of chicken breasts (*P* < 0.05). Specifically, reducing hardness by 22.63 %, 24.29 %, and 22.51 % in normal, moderate, and severe breast samples, respectively (Table 3). This improvement was particularly notable in moderate cases, suggesting optimal treatment efficacy for this severity level. This improvement might be attributed to ultrasound decreasing the particle size of myofibrillar protein. The enhancement mechanism involves ultrasound-induced reduction of myofibrillar protein (MP) particle size [59], leading to more stable and elastic oil-in-water (O/W) emulsion gels [60]. Furthermore, ultrasonic cavitation disrupts collagen fibrils and enhances protein solubilization [61], potentially counteracting the fibrotic characteristics of wooden breast. UAT treatment improves the eating quality of moderate breasts to a texture close to that of normal breasts, reducing the economic losses associated with wooden breast myopathy.

### 3.8 Collagen content

**Fig. 6:**
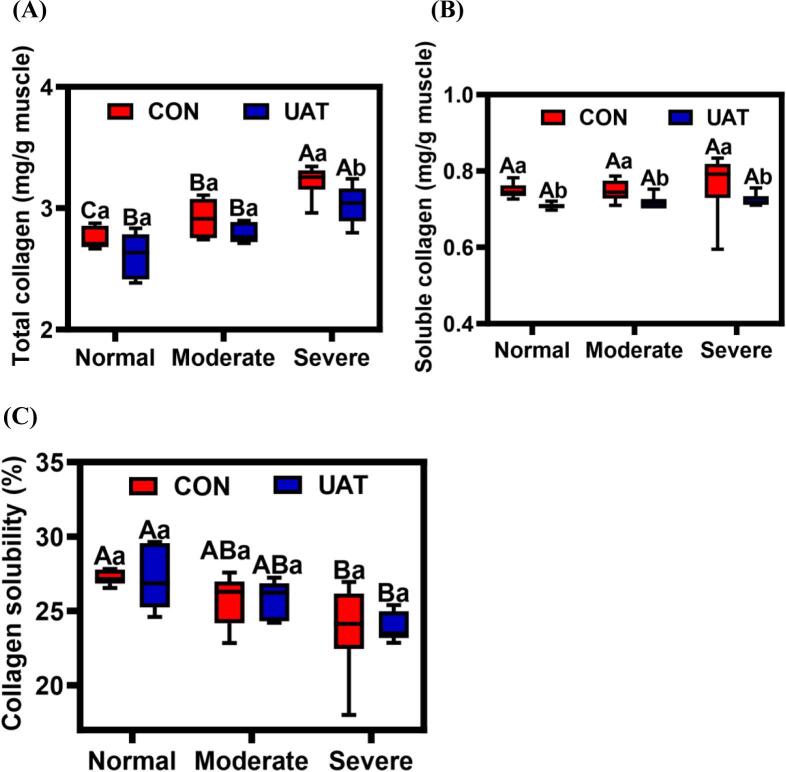
Effect of ultrasonic-assisted tumbling on collagen of wooden breast. C different letters indicate statistically significant difference between different degree of wooden breast (*P* < 0.05); a-c different letters indicate statistically significant difference between different treatment (*P* < 0.05); CON: control; UAT: ultrasonic-assisted tumbling (400 W, 20 kHz, 80 min); Normal: Normal breast; Moderate: Moderate wooden breast; Severe: Severe wooden breast.

Collagen is a hydroxyproline-rich protein that is a major component of the extracellular matrix [62]. As depicted in Fig. 6A, the wooden breast myopathy significantly affected total collagen content (*P* < 0.05), and collagen content increased with the severity of the myopathy. The higher total collagen content in wooden breast meat is primarily due to diffuse interstitial thickening of connective tissue, particularly in the endomysial and perimysial space [8]. Furthermore, the role of connective tissue in influencing meat tenderness depends largely on collagen dynamics. However, the collagen content of the breast decreased after ultrasound-assisted tumbling treatment. No significant difference (*P* > 0.05) was observed in collagen content between normal and moderate wooden breast tissues (2.60 mg/g vs 2.79 mg/g). Similar findings were reported by Du et al. [39], who indicated that ultrasonication decreased the total collagen content of chicken gizzards. This decrease may be related to the activation of matrix metalloproteinase zymogen, which is triggered by the mechanical and cavitation effects of ultrasound waves in the tissue. Once the signaling pathways were activated, this enzyme was capable of degrading connective tissues, resulting in collagen breakdown and reduced content [63]. Fig. 6B reveals that wooden breast myopathy did not have a significant effect on soluble collagen content (*P* > 0.05). In contrast, ultrasound-assisted tumbling treatment significantly reduced (from 0.77 mg/g to 0.72 mg/g) soluble collagen content (*P* < 0.05). Chang et al. [7] found that a 40 min low-frequency ultrasound treatment reduced collagen and soluble collagen content in beef while having a lesser effect on collagen solubility. From Fig. 6C, the collagen solubility content of severe wooden breast significantly decreased (*P* < 0.05). The increased collagen content is associated with decreased collagen solubility, contributing to the hardness and reduced tenderness of wooden breast meat. Nevertheless, the reduced solubility was mainly due to elevated levels of hydroxylysylpyridinoline cross-links, a type of mature cross-link between collagen molecules that is associated with collagen stability [12]. While ultrasound-assisted tumbling appeared to increase collagen solubility compared to the control group, the difference was not statistically significant (*P* > 0.05), suggesting limited practical impact on collagen stability. UAT degrades loosely cross-linked fibrils first rather than mature, HP-stabilized networks [11]. This selective removal aligns with UAT’s mechanical disruption of collagen architecture, which weakens fibril organization (shear force reductions) without dissolving stable cross-links. Since the collagen content is the primary determinant of WB meat toughness, by reducing collagen quantity and reorganizing fibril networks [13], UAT achieves tenderness improvements comparable to enzymatic methods while avoiding over-softening defects. These findings suggest that the accumulation of collagen and its cross-linking are key factors underlying the development of wooden breast myopathy and the deterioration of meat quality. Effectively modulate collagen content and solubility, potentially leading to improved meat quality in the poultry industry.

## 4 Conclusion

Wooden breast myopathy affects the quality characteristics of chicken breast meat, especially in terms of water-holding capacity and tenderness. In this study, ultrasonic-assisted tumbling treatment at 400 W for 80 min significantly improved the marinating absorptivity, water-holding capacity, and textural properties of wooden breast meat. After ultrasonic-assisted tumbling treatment, the tenderness and water-holding capacity of wooden breast meat were markedly enhanced, even reaching the quality level of untreated normal breast muscle. The solubility of salt-soluble proteins increased, while the content of collagen and soluble collagen decreased, although the solubility of collagen did not change significantly. The combined effects of ultrasonic cavitation and mechanical action disrupt the muscle tissue, release salt-soluble proteins to the meat surface, and enhance protein-water interactions, thereby improving tenderness and water-holding capacity. These findings suggest that ultrasonic-assisted tumbling can effectively improve the quality of WB meat, reduce treatment time for conventional tumbling methods. Moreover, UAT reduces marination time and enhances product uniformity, and minimized waste, offering a promising technology to enhance meat quality.

## CRediT authorship contribution statement

**Yanyan Lu:** Writing – review & editing, Writing – original draft, Visualization, Validation, Supervision, Software, Resources, Project administration, Methodology, Investigation, Funding acquisition, Formal analysis, Data curation. **Zhenyang Wu:** Validation, Software, Data curation, Conceptualization. **Tianjiao Bian:** Validation, Data curation, Conceptualization. **Xue Zhao:** Supervision, Resources, Conceptualization.

## Declaration of competing interest

The authors declare that they have no known competing financial interests or personal relationships that could have appeared to influence the work reported in this paper. We would like to submit the enclosed manuscript entitled “Ultrasonic-Assisted Tumbling Improves Water Retention and Tenderness of Wooden Breast Chicken Meat”, which we wish to be considered for publication in “Ultrasonics Sonochemistry”. No conflict of interest exits in the submission of this manuscript, and manuscript is approved by all.
